# Evaluation of the Kodak Ektachem DT60 analyser for sodium, potassium, glucose and urea

**DOI:** 10.1155/S1463924687000269

**Published:** 1987

**Authors:** Gwendolen J. Ayers, Caroline Sarah Sherab Rumjen, Terence F. Woods, David Burnett

**Affiliations:** Department of Clinical Biochemistry St. Albans City Hospital Normandy Road Hertfordshire St. Albans UK


**Journal of Automatic Chemistry**/*Journal of Clinical Laboratory Automation*, **Vol. 9, No. 3 (July–September 1987), pp. 119–124**

### Evaluation of the Kodak Ektachem DT60 analyser for sodium, potassium, glucose and urea

**Gwendolen J. Ayers, Caroline Sarah Sherab Rumjen, Terence F. Woods and David Burnett***

*Department of Clinical Biochemistry, St. Albans City Hospital, Normandy Road, St. Albans, Hertfordshire, UK*

*The Kodak Ektachem DT60/E analyser is a desk-top analyser which uses thin-film 'dry' chemistry technology to perform colorimetric and potentiometric analyses. The analyser is simple to operate and the slides are claimed to be stable for up to one year if stored at 4 °C. Calibration is said to be required only once every three months. Results presented in this paper substantiate these claims and show that the Ektachem DT60/E produces results which are comparable in terms of bias and precision with currently used methods for sodium, potassium, urea and glucose. The instrument might have an application within the laboratory for emergency/on-call work as well as for out-of-laboratory testing.*

### Introduction

There have been several reports of the performance characteristics of high-throughput automatic analysers using Kodak thin-film technology [1–5]. A small, manually operated analyser using the same technology, designed for use in doctor's office laboratories, has recently become available. The analyser consists of a microcomputer/reflectance photometer (Ektachem DT60) for colorimetric analyses and an optional electrometer (DTE module) for potentiometric analyses which are performed using a slide consisting of a pair of identical direct ion-selective electrodes. The simple mode of operation, recalibration frequency of three months, stability of the slides, potential variety of tests available and small sample requirement suggest that the instrument may be useful in the hospital laboratory for urgent analyses, especially out-of-hours requests, for paediatric work and for analyses which are requested relatively rarely.

In the present paper, performance characteristics (imprecision, bias against various comparative methods, linearity, interferences) and various practical aspects (stability of slides, effect of multiple operators, effect of incubation time for electrolyte analyses) of the Ektachem DT60/E are described for four of the most commonly requested 'urgent' tests, i.e. sodium, potassium, urea and glucose. The results are compared with manufacturers' claims and performance criteria based on biological variation [6].

* To whom correspondence and requests for reprints should be addressed.

### Materials and methods

### *Ektachem DT60/E*

#### *Slides*

Colorimetric slides consist of reagents prepared in thin layers on a film base and mounted in a plastic mount 28 × 33 mm. A bar code on the upper surface identifies the analyte and the generation (batch) number of the slide. The glucose slide uses glucose oxidase/peroxidase and 4-aminoantipyrine to give a red colour. The urea slide consists of urease, a semi-permeable membrane and a dye which turns blue/black in the presence of ammonium ions. Above the reagent layers is a cellulose/titanium oxide layer which spreads the sample evenly over the reagent area (so that the volume of sample applied is not critical). The colour developed in the slide is read from below, through the transparent film base, using a fibre optic reflectance system (FORS). The titanium oxide in the spreading layer provides a highly reflective surface.

Potentiometric slides consist of a pair of direct reading ion-selective electrodes, linked by a paper bridge, mounted in a plastic mount 28 × 33 mm. The bar code identifying the analyte and slide generation number is on the lower surface as the electrometer makes contact with the exposed silver areas of the electrodes on the upper surface of the slide.

The slides are individually foil-wrapped and packed in boxes of 25. The manufacturer claims that the slides are stable for more than one year if kept at 4 °C.

### *Instrumentation*

#### *Hardware and operation*

The DT60 reflectance photometer is approximately 0·5 m wide by 0·35 m deep. A touch-sensitive keyboard enables numerical data to be entered and special functions to be selected. During operation the operator has the option to assign an identification number of up to 10 characters to the sample about to be analysed. Above the keyboard is a two-line display which gives the operational status of the instrument and guides the operator through the calibration and operation procedures.

At the right-hand side of the instrument is the slide loader and slide-spotting station. When a colorimetric slide is loaded a bar code identifies the analyte and generation number of the slide. 10 µl serum or plasma is dispensed onto the slide using the motorized pipette supplied, following the prompt from the display screen. An optical sensor detects the change in reflectance of the spreading layer caused by wetness. The slide is then transferred automatically to the incubator. After 313 s at 37 °C the slide is moved to the reading station, the colour developed is read by the fibre optic reading system (FORS) and the slide then ejected into a waste compartment at the back of the instrument. The incubator can hold up to six slides at a time.

The reflectance reading is converted to a concentration reading by the microcomputer using stored calibration parameters and the result is printed by the thermal printer at the left-hand side of the analyser.

The DTE module is 0·14 m wide by 0·35 m deep. Disposable sample cups with two wells of unequal size are provided. The sample for analysis is placed in the larger well and electrolyte reference fluid is placed in the other. The asymmetry of the wells ensures the correct orientation of sample and reference fluid in the pick-up station. A dual, manually operated pipette is used to aspirate and dispense equal volumes (10 μl) for sample and reference fluid. Locators on the pipette pick-up and slide-spotting stations ensure the correct orientation of the pipette during each operation. When the slide has been spotted the fluids spread towards each other along a paper bridge and after allowing time for the formation of a stable liquid junction, the potential difference between the two half cells is measured. The temperature is maintained at 25 °C. The microcomputer calculates the sample concentration from the measured voltage using stored calibration parameters and the result is printed as for colorimetric tests.

The incubator capacity of six slides and an incubation time of 313 s gives a claimed throughput of 65 colorimetric slides/h. The read time of 180 s for potentiometric slides (analysed one at a time) gives a claimed throughput of 15 slides/h. The DT60 and DTE modules can be used simultaneously.

*Calibration*

Calibration is achieved by analysing bovine serum-based calibration fluids in the same way as patient samples. Calibration models and assigned values for each analyte are stored in the calibration data module (CDM) of the microcomputer. A new CDM is supplied when the generation number of the slides is changed and new calibrator values may be needed. The recommended procedure is to analyse calibrators at three analyte concentrations singly for colorimetric tests and two analyte concentrations in duplicate for potentiometric tests.

*Comparative methods*

Sodium and potassium results from the DT60/E were compared with results obtained by flame photometry using an Instrumentation Laboratories IL543 and the Technicon SMAIIC. Two direct reading ion-selective electrode instruments NOVA-1 (Clandon Scientific) and Corning 614 (Ciba-Corning Diagnostics Ltd) were also used.

Comparative methods for urea were the Technicon SMAIIC (diacetyl monoxime) and the Beckman BUN 2 Analyser. Glucose was measured by a glucose oxidase method (BCL-GOD/PAP, Boehringer Corporation [London] Ltd) using Technicon Auto-analyser I (AA1) instrumentation and also by the Yellow Springs Instruments Glucose Analyser (YSI, Clandon Scientific).

With the exception of the AA1 method for glucose, which was calibrated with standards prepared in the laboratory, all instruments were calibrated with fluids provided by the manufacturers for that particular instrument and using the manufacturer's recommended protocols.

*Materials*

*Patient samples*

Samples were selected from the results of an initial analysis (SMAIIC or YSI) so that the distributions of analyte concentrations used were those recommended in the *NCCLS Protocol* (PSEP-4) [7]. In order to comply with the *Protocol*, samples for urea and glucose were stored deep frozen until analysed. All measurements on any individual sample were made on the same day.

*Quality-control pools*

During the evaluation (April–May 1985, Period 1) a number of bovine serum quality-control pools were analysed in duplicate each day (Kodak DT Control Lot 1-Kodak; Autoset M, SRVS-J6, SRVS-L6, SRVS-I6, Wellcome Diagnostics). The materials were also run over a longer period (November 1984–March 1985, Period 2).

*External quality assessment samples*

A series of 10 pools were obtained from the Wales External Quality Assessment Scheme. These were supplied in liquid form and were prepared by diluting a human serum pool with high analyte concentrations with a similar pool having low analyte concentrations. The concensus mean value (all methods) after exclusion of outliers was taken as the assigned value for each analyte.

### Results

*Performance of comparative methods*

The linearity of response for all methods was confirmed by inspection of plots of observed against expected concentration for the Wales EQAS external assessment materials. Linear regression statistics for these plots are shown in table 1. The SMAIIC was not included in this part of the study as its performance was previously judged to be satisfactory on the basis of internal quality-control and performance in external quality assessment schemes (WEQAS, NEQAS, Wellcome Quality Control Programme).

*Precision and stability of calibration*

Estimates of overall and within-batch coefficients of variation are shown for various control pools in table 2. The coefficients of variation for glucose and urea for Period 2 (five months) were larger than those for Period 1 (two months). This was probably due to deterioration of

*Table 1. Performance of the DT60 and comparative methods on material from the Wales EQAS. Linear regression statistics for observed (y) against expected value (x) for external assessment materials (N = 10).*

| Analyte | Range of values mmol/l | Method | Slope | Intercept | $S_{yx}$ | $x$ | $y$ | $Sd_x/S_{yx}$ | $r$ |
|---|---|---|---|---|---|---|---|---|---|
| Sodium | 105–154 | DT60 | 1·00 | −1·30 | 1·09 | 129·6 | 128·3 | 15·00 | 0·998 |
| | | IL543 | 1·04 | −3·26 | 1·80 | 129·6 | 131·5 | 9·07 | 0·995 |
| | | NOVA-1 | 1·19 | −24·39 | 0·75 | 129·6 | 129·8 | 21·84 | 0·999 |
| | | Corning 614 | 1·03 | −6·33 | 0·56 | 129·6 | 127·1 | 29·17 | 0·999 |
| Potassium | 1·8–7·2 | DT60 | 0·96 | 0·17 | 0·05 | 4·5 | 4·5 | 19·54 | 0·999 |
| | | IL543 | 1·04 | −0·13 | 0·07 | 4·5 | 4·5 | 25·09 | 0·999 |
| | | NOVA-1 | 0·92 | 0·17 | 0·06 | 4·5 | 4·3 | 30·54 | 0·999 |
| | | Corning 614 | 0·96 | 0·00 | 0·05 | 4·5 | 4·3 | 37·23 | 1·000 |
| Glucose | 2·02–19·26 | DT60 | 1·04 | −0·15 | 0·04 | 10·6 | 10·9 | 153·45 | 1·000 |
| | | YSI | 1·03 | −0·10 | 0·06 | 10·6 | 10·8 | 93·45 | 1·000 |
| | | AA1 | 0·96 | −0·20 | 0·14 | 10·6 | 10·0 | 40·38 | 1·000 |
| Urea | 12·17–19·38 | DT60 | 1·00 | −1·30 | 0·36 | 10·8 | 9·6 | 16·26 | 0·998 |
| | | Beckman | 1·07 | −0·58 | 0·14 | 10·8 | 11·0 | 40·29 | 1·000 |

*Table 2. Kodak Ektachem DT60 – imprecision with pooled sera.*

| Analyte | Material | Assigned value mmol/l | Period 1 (April–May 1985) Overall CV % | (n) | Period 1 Within batch CV % | (n) | Period 2 (November 1984–March 1985) Overall CV % | (n) | Period 2 Within batch CV % | (N) |
|---|---|---|---|---|---|---|---|---|---|---|
| Sodium | DT60 Control | 150 | 0·70 | (18) | 0·39 | (9) | 0·76 | (103) | 0·63 | (51) |
| | Autoset M* | — | 1·13 | (24) | 0·88 | (12) | — | | — | |
| | SRVS I6 | 142 | — | | — | | 1·45 | (157) | 0·82 | (78) |
| | SRVS J6 | 117 | — | | — | | 1·04 | (148) | 0·85 | (73) |
| Potassium | DT60 Control | 6·0 | 1·91 | (14) | 1·58 | (7) | 1·25 | (103) | 1·22 | (51) |
| | Autoset M | — | 2·09 | (20) | 0·73 | (10) | — | | — | |
| | SRVS I6 | 3·92 | — | | — | | 1·79 | (153) | 1·17 | (77) |
| | SRVS J6 | 3·11 | — | | — | | 1·95 | (151) | 1·49 | (78) |
| Urea | DT60 Control | 7·05 | 1·80 | (20) | 1·26 | (10) | 3·50 | (101) | 2·51 | (50) |
| | Autoset M | — | 2·21 | (20) | 1·97 | (10) | — | | — | |
| | SRVS I6 | 11·10 | — | | — | | 3·67 | (150) | 2·25 | (76) |
| | SRVS J6 | 17·50 | — | | — | | 3·43 | (151) | 2·28 | (76) |
| Glucose | DT60 Control | 6·3 | 2·26 | (34) | 1·00 | (17) | 3·80 | (101) | 1·29 | (50) |
| | Autoset M | — | 0·87 | (32) | 0·71 | (16) | — | | — | |
| | SRVS I6 | 6·38 | — | | — | | 4·75 | (151) | 1·25 | (76) |
| | SRVS J6 | 12·00 | — | | — | | 3·26 | (151) | 1·11 | (76) |

* Autoset M is unassayed.

the control materials, which were stored deep-frozen in aliquots, over the longer time. Precision profiles for duplicate (within-batch) measurements on patient samples are shown in table 3.

*Assessment of bias*

Plots of values obtained for patient samples on the DT60 against comparative method values were found to be linear on visual inspection. The linear regression statistics after exclusion of outliers (difference between pair of results exceeded 3·5 $S_{yx}$ [6]) are shown in table 4. With the possible exception of the data for sodium, the range of concentrations in the samples analysed was sufficiently wide in relation to the imprecision of the test and comparative methods ($SD_x/S_{xy} > 7$) to justify the use of conventional linear regression statistics[6]. In the case of sodium this method of calculation would cause an under-estimation of the slope and consequent over-estimation of the $y$-intercept. The slopes and intercepts shown in table 4 for sodium were therefore calculated by the Deming method.

All comparisons except that for urea on the SMAIIC show evidence of proportional and constant error as judged by linear regression analysis. However, the mean bias ($y - x$, table 4) was considered to be of practical importance only for glucose (+0·88 mmol/l against AA1, and +0·67 mmol/l against YSI) and possibly sodium

*Table 3. Precision on patient samples.*

| Analyte | Method | Number of samples | Percentage of duplicates with CV of 0–1·5% | 1·5–3% | 3–4·5% | 4·5–6% | >6% |
|---|---|---|---|---|---|---|---|
| Sodium | DT60 | 70 | 98·6 | 1·4 | — | — | — |
| | IL543 | 70 | 100·0 | — | — | — | — |
| | NOVA-1 | 68 | 100·0 | — | — | — | — |
| | Corning 614 | 70 | 100·0 | — | — | — | — |
| Potassium | DT60 | 70 | 65·7 | 31·4 | 2·9 | — | — |
| | IL543 | 70 | 91·4 | 7·1 | 1·5 | — | — |
| | NOVA-1 | 68 | 82·4 | 17·6 | — | — | — |
| | Corning 614 | 70 | 90·0 | 10·0 | — | — | — |
| Glucose | DT60 | 100 | 95·0 | 5·0 | — | — | — |
| | AA1 | 100 | 69·0 | 25·0 | 3·0 | 1·0 | 2·0 |
| | YSI | 100 | 86·0 | 11·0 | 2·0 | 1·0 | — |
| Urea | DT60 | 74 | 77·0 | 17·6 | 1·4 | 2·7 | 1·4 |
| | Beckman | 75 | 42·7 | 18·7 | 12·0 | 12·0 | 14·6 |

*Table 4. Linear regression statistics for Ektachem DT60 (y axis) against various comparative methods (x axis).*

| Analyte | Comparative method | $N$ | Slope | Intercept | $Syx$ | x | y | $SD_x/S_{yx}$ | $r$ | Number of outliers $>3{\cdot}5\,S_{yx}$ |
|---|---|---|---|---|---|---|---|---|---|---|
| Sodium | SMAIIC | 79 | 0·92 | 9·5 | 2·37 | 137·4 | 136·9 | 4·9 | 0·977 | 1 |
| | IL543 | 84 | 0·95 | 5·4 | 2·39 | 138·7 | 137·2 | 4·7 | 0·976 | 1 |
| | Nova-1 | 80 | 0·87 | 4·9 | 2·16 | 141·8 | 138·3 | 5·4 | 0·977 | 0 |
| | Corning 614 | 84 | 0·93 | 8·9 | 2·21 | 137·6 | 136·9 | 5·1 | 0·979 | 0 |
| Potassium | SMAIIC | 65 | 0·95 | 0·46 | 0·11 | 4·5 | 4·7 | 9·3 | 0·994 | 0 |
| | IL543 | 70 | 0·93 | 0·47 | 0·09 | 4·6 | 4·7 | 10·5 | 0·995 | 0 |
| | Nova-1 | 68 | 0·96 | 0·22 | 0·07 | 4·7 | 4·7 | 14·5 | 0·998 | 0 |
| | Corning 614 | 70 | 0·97 | 0·27 | 0·07 | 4·6 | 4·7 | 12·6 | 0·997 | 0 |
| Glucose | AA1 | 100 | 1·05 | 0·57 | 0·26 | 6·75 | 7·63 | 14·6 | 0·998 | 0 |
| | YSI | 100 | 1·06 | 0·22 | 0·27 | 6·96 | 7·63 | 13·6 | 0·998 | 0 |
| Urea | SMAIIC | 73 | 1·02 | 0·21 | 0·59 | 9·36 | 9·79 | 10·1 | 0·995 | 2 |
| | Beckman | 73 | 0·95 | 0·83 | 0·51 | 9·41 | 9·71 | 12·3 | 0·996 | 1 |

(−1·5 mmol/l against IL543 and 3·5 mmol/l against NOVA-1). However, from the analysis of external assessment material (table 1) there is evidence that the comparative methods for glucose had a degree of negative bias compared to consensus means and that sodium on the IL543 and NOVA-1 had positive bias.

### *Interferences*

Lithium at concentrations of up to 5 mmol/l did not interfere with sodium or potassium measurements by any of the methods used. Paracetamol at concentrations up to 500 mg/l did not interfere with glucose measurement on the DT60 or autoanalyser method. Interference in the YSI method is well documented [8] and special membranes which reduce the effect of paracetamol can be purchased.

### *Effect of operator*

Nineteen members of staff from three hospitals were given a short course of instruction (30–60 min) on the use of the DT60; the course included an explanation of the calibration procedure and some practical experience. Results obtained by these operators for duplicate determination of urea in two quality-control pools are shown in figure 1. Similar results, i.e. no significant operator dependence, were found for other analytes. Ability to use the pipettes correctly is a prime requisite for using the instrument and during the training period attention was paid to familiarization with the motorized (DT60) and dual-tipped manual (DTE) pipettes.

### *Stability of slides*

The manufacturers recommend that slides are stored at 4 °C and time must be allowed for them to reach room temperature before use. This delay may be inconvenient and could result in slide wastage. The effect of storage conditions was, therefore, investigated. Slides were removed from the deep freeze, allowed to come to room temperature then kept for alternate seven hour periods at room temperature and 17-hour periods at +4 °C for four days. Using one control material analyses were performed at the beginning (0900 h) and end (1600 h) of each seven hour period. No deterioration was noted over the four-day period. The results for glucose and urea are shown in figure 2.

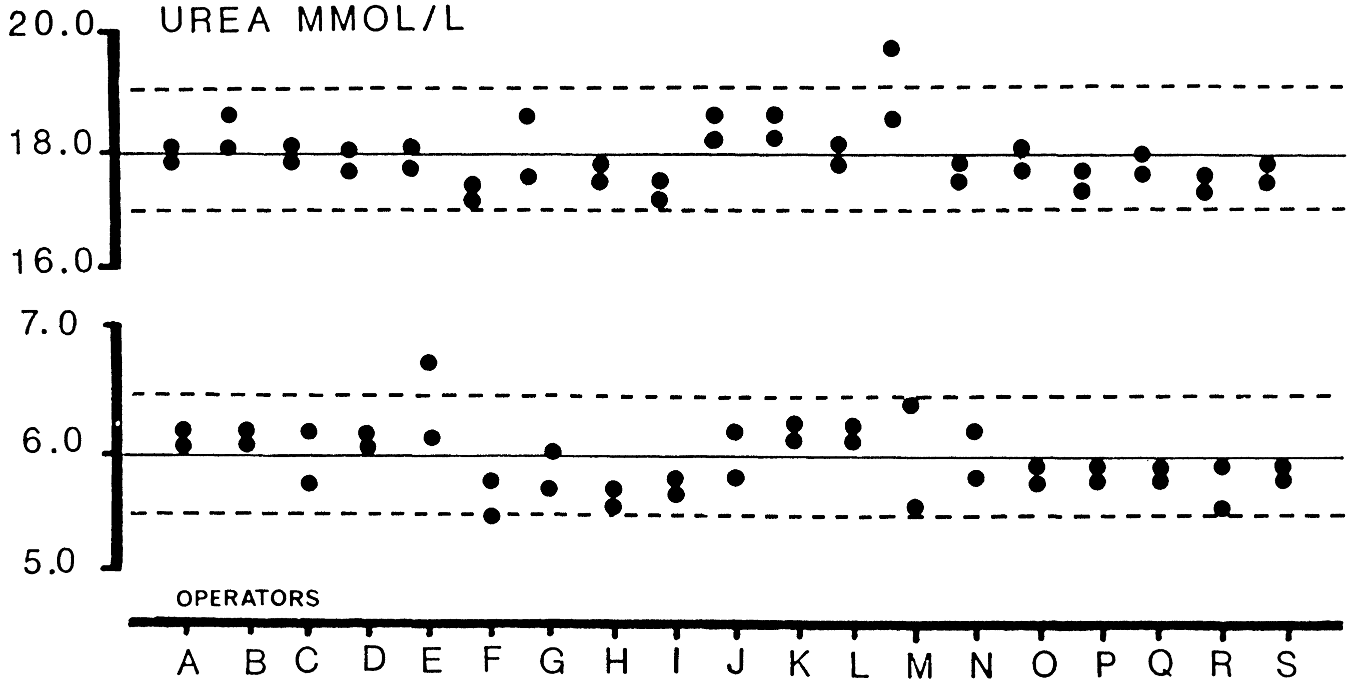

*Figure 1. Results obtained by multiple operators (A-S) on two QC samples using Ektachem urea slides.*

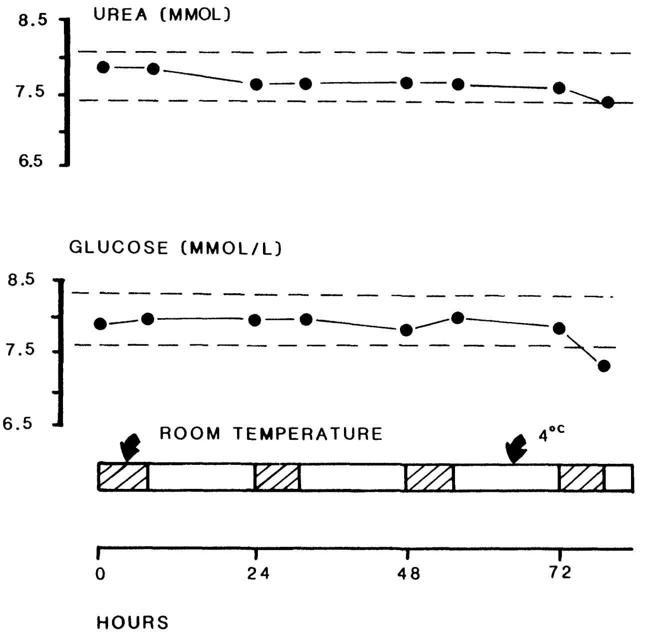

*Figure 2. Effect of storage on performance of Ektachem urea and glucose slides.*

In a second experiment slides for all analytes were found to be stable when kept for 72 h at room temperature (21–25 °C) or at 37 °C.

### *Effect of incubation time of sodium and potassium results*

No significant differences were found between the means for 30 patient samples analysed in duplicate with incubation times of 180, 90 or 60 s (table 5).

### *Other aspects of practicability*

The manufacturer's claim >95% of slides yield reportable patient results. In our study >98% yielded reportable results.

In five months two service calls were necessary due to (*a*) a problem with the printer following a change of paper; (*b*) malfunction of the electronic pipette. The electronic pipette is a sealed unit not intended for servicing by the user. Kodak policy is to replace the pipette and overhaul the returned unit in their workshop. The printer problem was related to operator inexperience.

### Discussion

The manufacturer's claim for dynamic ranges of 95–215 mmol/l for sodium, 1–9 mmol/l for potassium, 1–25 mmol/l for glucose and 2–36 mmol/l for urea was supported by our data. The reproducibility of the DT60 with patient samples was better than that of the comparative methods for urea and glucose but slightly poorer for sodium and potassium. The precision was adequate to meet the relatively strict targets for precision derived from biological variability [6] for potassium (target CV 2·2%), urea (target CV 6·2%), glucose (target CV 2·2%) but not for sodium (target CV 0·4%). However, the comparative methods cannot achieve the target for sodium either.

In the method comparison experiments evidence of proportional and/or constant errors was found for all analytes. This was due in part to bias in the comparative methods.

There is of course a fundamental difference in what is being measured by the flame photometric (SMA, IL543)

*Table 5. Effect of incubation time on sodium and potassium results.*

| Incubation time (seconds) | Sodium Mean* mmol/l | Sodium 't' test | Potassium Mean* mmol/l | Potassium 't' test |
|---|---|---|---|---|
| 180 | 134·0 | | 4·38 | |
| 90 | 133·6 | 1·57 | 4·37 | 0·76 |
| 60 | 134·2 | 0·81 | 4·40 | 0·96 |

* Mean value for 30 patient samples analysed in duplicate.
No significant differences between means found in paired 't' tests.

and direct ion-selective electrode (ISE) (Kodak, Corning 614, NOVA-1) methods and various recommendations have been made as to what should be reported and how the direct ISE instruments should be calibrated [11–18]. The high correlations and good precision found with the DT60 indicate that it would be possible to improve comparability with modified calibration procedures.

The results obtained were consistent with the manufacturer's claim that calibration at three-monthly intervals was adequate and also showed that relatively inexperienced operators could produce satisfactory results. For a single sample, sodium, potassium, urea and glucose results could be available in seven minutes. The incubation time of three minutes per electrolyte measurement is the limiting factor but our results indicate that this could be reduced. The instrument was reliable as judged by the low frequency of service calls and small percentage of wasted slides. The absence of liquid reagents, containment of specimen within the disposable slide and enclosure of all moving parts are conducive to electrical, microbiological and mechanical safety.

The simple mode of operation, infrequent need for calibration and stability of the slides make this machine suitable for low volume work, particularly that performed urgently or outside laboratory working hours. The small sample volume required means that the analyser can be readily used for paediatric as well as adult specimens.

### References

1. Burnett, D., Barbour, H. M., Woods, T. F., Vassault, A., Sebille, L. *et al.*, A multicentre evaluation of the Kodak EKTACHEM GLU/BUN analyzer using NCCLS guidelines and other approaches. *Journal of Clinical Chemistry and Clinical Biochemistry,* **20** (1982), 207–215.
2. Costello, P., Kubasik, N., Brody, B., Sine, H., Bertsch, J. and D'Sousa, J. P., Multilayer film analysis: evaluation of ion-selective electrolyte slides. *Clinical Chemistry,* **29** (1983), 129–132.
3. Cate, J. C., Hedrick, R., Gouchoroff, D. *et al.*, Operating and performance characteristics of the EKTACHEM 400 analyzer. *Journal of Clinical Laboratory Automation* (1984), 43–48.
4. Kadinger, C. L. and O'Kell, R. T., Clinical evaluations of Eastman Kodak's EKTACHEM 400 analyzer. *Clinical Chemistry,* **29** (1983), 498–501.
5. Knoll, E., Hafner, F. W., Dettmer, K., Wesser, N. The determination of calcium, glucose, urea and uric acid using the Kodak Ektachem multilayer film technology: an evaluation. *Journal of Clinical Chemistry Biochemistry,* **20** (1982), 491–498.
6. Fraser, C. G., Desirable performance standards for clinical chemistry tests. *Advances in Clinical Chemistry* (Academic Press, 1983).
7. *NCCLS Proposed Standard PSEP-4.* Protocol for estabishing performance claims for clinical chemical methods. Comparison of methods experiment. (National Committee for Clinical Laboratory Standards, 771E Lancaster Avenue, Villanova, Pennsylvania 29085, 1979).
8. Burrin, J. M. and Price, C. P., Measurement of blood glucose. *Annals of Clinical Biochemistry,* **22** (1985), 327–342.
9. Annan, W., Kirwan, N. A., Robertson, W. S., Teasdale, P. R. and Ager, B. P., An evaluation of the NOVA-1 ion selective electrode analyser for Na+ and K+ determination. *Journal of Automatic Chemistry,* **2** (1980), 212–218.
10. Ladenson, J. H., Direct potentiometric analysis of sodium and potassium in human plasma: evidence for association with a non-protein, protein-associated substance. *Journal of Laboratory Clinical Medicine,* **25** (1977), 654–665.
11. Levy, G. B., Determination of sodium with ISEs. *Clinical Chemistry,* **27** (1981), 1435–1438.
12. Maas, A. H. J. and Siggard-Andersen, D., Ion-selective electrodes for sodium and potassium: a new problem of what is being measured and what should be reported. *International Federation of Clinical Chemistry News,* **2** (1982), 5–6.
13. Fogh-Anderson, N., Wimberley, P. D., Thode, J., Siggard-Andersen, D., Determination of sodium and potassium with ISEs. *Clinical Chemistry,* **30** (1984), 433–436.
14. Worth, H. G. J., Clinical ions – ISE or flame. *Laboratory Practice* (April 1984), 15–16.
15. Buckley, B. M., Broughton, P. M. G., Russell, L. J., Carter, T. J. N. New ways with old ions. *Annals of Clinical Biochemistry,* **21** (1984), 75–77.
16. Worth, H. G. J., A comparison of the measurement of sodium and potassium by flame photometry and ion-selective electrode. *Annals of Clinical Biochemistry,* **22** (1985), 343–350.
17. Burns, R. F. and Russell, L. J., Ion-selective electrode technology: an overview. In *Clinical Chemistry nearer the Patient.* Marks V and Alberti K. G. M. M. (eds). (Churchill Livingstone, Edinburgh, 1985), p. 121–130.
18. Maas, A. H. J., Siggard-Andersen, O., Weisberg, H. F. and Zijlstra, W. G., Ion-selective electrodes for sodium and potassium: a new problem of what is measured and what should be reported. *Clinical Chemistry,* **31** (1985), 482–485.

